# Oil-In-Water Microemulsions as Hosts for Benzothiophene-Based Cytotoxic Compounds: An Effective Combination

**DOI:** 10.3390/biomimetics3020013

**Published:** 2018-06-16

**Authors:** Ioanna Theochari, Vassiliki Papadimitriou, Demetris Papahatjis, Nikos Assimomytis, Efthimia Pappou, Harris Pratsinis, Aristotelis Xenakis, Vasiliki Pletsa

**Affiliations:** 1 Institute of Biology, Medicinal Chemistry & Biotechnology, National Hellenic Research Foundation, 48 Vassileos Constantinou Avenue, 11635 Athens, Greece; jtheohari@eie.gr (I.T.); vpapa@eie.gr (V.Pa.); dpapah@eie.gr (D.P.); nassim@eie.gr (N.A.); epappou@eie.gr (E.P.); arisx@eie.gr (A.X.); 2 Department of Biochemistry and Biotechnology, School of Health Sciences, University of Thessaly, Viopolis, 41500 Larissa, Greece; 3 Laboratory of Cell Proliferation and Ageing, Institute of Biosciences and Applications, National Centre of Scientific Research “Demokritos”, 11635 Athens, Greece; hprats@bio.demokritos.gr

**Keywords:** microemulsions, drug delivery, electron paramagnetic resonance, DNA replication fork, mechanisms of cell death, chemotherapy

## Abstract

Targeted delivery of chemotherapeutics in order to overcome side effects and enhance chemosensitivity remains a major issue in cancer research. In this context, biocompatible oil-in-water (O/W) microemulsions were developed as matrices for the encapsulation of DPS-2 a benzothiophene analogue, exhibiting high cytotoxicity in various cancer cell lines, among them the MW 164 skin melanoma and Caco-2 human epithelial colorectal adenocarcinoma cell lines. The microemulsion delivery system was structurally characterized by dynamic light scattering (DLS) and electron paramagnetic resonance (EPR) spectroscopy. The effective release of a lipophilic encapsulated compound was evaluated via confocal microscopy. The cytotoxic effect, in the presence and absence of DPS-2, was examined through the thiazolyl blue tetrazolium bromide (MTT) cell proliferation assay. When encapsulated, DPS-2 was as cytotoxic as when dissolved in dimethyl sulfoxide (DMSO). Hence, the oil cores of O/W microemulsions were proven effective biocompatible carriers of lipophilic bioactive molecules in biological assessment experiments. Further investigation through fluorescence-activated cell sorting (FACS) analysis, comet assay, and Western blotting, revealed that DPS-2, although non-genotoxic, induced S phase delay accompanied by cdc25A degradation and a nonapoptotic cell death in both cell lines, which implies that this benzothiophene analogue is a deoxyribonucleic acid (DNA) replication inhibitor.

## 1. Introduction

Over the last ten years, research groups worldwide have focused more attention on the development and structural characterization of safe biocompatible, soft nanosized systems to create materials with potential application in the field of targeted drug delivery. More specifically, the use of colloidal delivery systems, such as liposomes, polymers, nanoparticles, emulsions and microemulsions, has increased due to their ability to affect the pharmacokinetics and biodistribution of drugs. In particular, microemulsions have attracted increased interest as potential drug carriers due to their promising applications in the field of cancer chemotherapy [1,2,3].

Chemotherapy is a type of cancer treatment that uses cytotoxic drugs to kill cancer cells by stopping or slowing their growth. In fact, chemotherapy not only kills fast-growing cancer cells, but also kills or slows the growth of healthy cells, causing important side effects. In this respect, targeted delivery of chemotherapeutics using microemulsions could prevent or control side effects and enhance chemosensitivity. As a result, drug toxicity could be decreased and tumor response considerably increased [1,2].

Microemulsions are low viscous, optically transparent, thermodynamically stable dispersions of oil and water stabilized by surfactant molecules. They are isotropic in nature with typical droplet diameters in the size range of 10–100 nm. Microemulsions can be classified into three main categories according to the ratios of the dispersed phase in relation to the dispersion medium: oil-in-water (O/W), water-in-oil (W/O), and bicontinuous [4,5,6]. Pharmaceutical microemulsions as drug delivery systems are becoming very important due to their potential to incorporate a wide range of drug molecules, both hydrophilic and hydrophobic. Furthermore, microemulsions could improve the bioavailability and also allow controlled release of the encapsulated drugs [7,8]. The main aim of this study was the development and characterization of O/W microemulsions based on safe materials as carriers of the benzothiophene analogue DPS-2 and the in vitro biological assessment of its delivery in human cell lines.

**Figure 1:**
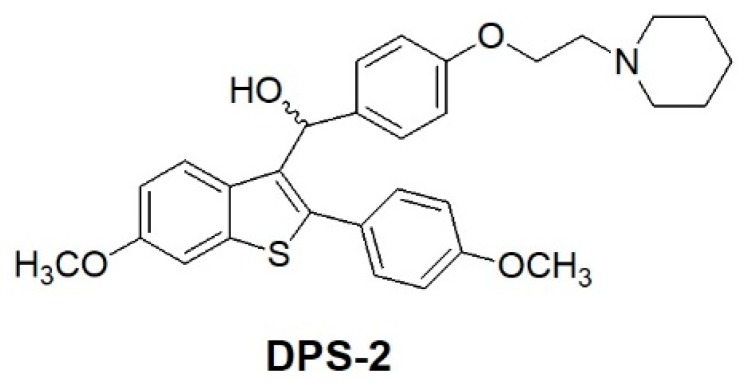
Chemical structure of DPS-2.

The benzo[*b*]thiophene scaffold is a structure widely used in drug discovery as a starting material for the synthesis of larger bioactive molecules capable of acting as antimicrobial, anti-inflammatory, and anticancer agents [9]. Benzothiophene compounds of the general formula, depicted in Figure 1, have been previously described as useful medications for the treatment of various medical indications associated with post-menopausal syndrome, uterine fibroid disease, endometriosis and aortal smooth muscle cell proliferation [9,10]. The encapsulated compound (DPS-2) is a pharmaceutical benzothiophene analogue initially designed to inhibit the BRAF^V600E^ oncogenic kinase [11,12,13]. Although it has not been proven a specific BRAF^V600E^ inhibitor, DPS-2 exhibited significant cytotoxic activity in human colon cancer cell lines [14]. Thus, further investigation was conducted targeting the MW 164 skin melanoma and Caco-2 human epithelial colorectal adenocarcinoma cell lines, both bearing mutated BRAF^V600E^ and p53 [15,16] and being sensitive to the compound. Moreover, in the course of the study, a major question addressed was whether DPS-2, in view of its high cytotoxicity, could serve as a lead compound to develop a novel chemotherapeutic agent. Besides apoptosis or necrosis, chemotherapeutic agents may activate several other modes of cell death such as autophagy, senescence, and/or mitotic catastrophe [17,18,19]. Understanding the mechanism through which a given chemotherapy affects all the signaling pathways involved in cell survival/death is critical for developing effective, targeted therapeutics. Thus the molecular basis underlying the decision-making process is currently the subject of intense investigation [20].

Pharmaceutical microemulsions are comprised of biocompatible, safe, and clinically acceptable ingredients. To lower interfacial tension and enable mutual solubilization of the two immiscible liquids, nonionic surfactants at appropriate concentrations, should be used. In general, the concentrations of surfactants should be kept low to ensure the development of mild and nonaggressive systems. In the case of O/W microemulsions, the oil phase must be chosen appropriately to ensure drug solubility and effective drug transportation to the cells. In the present study triacetin, a triester of glycerol and acetic acid that is able to solubilize lipophilic drugs and also facilitate the formation of microemulsions with desired characteristics, was selected. Polysorbate 80, a widely used surfactant in medical, cosmetic and food preparations, was used as surfactant avoiding the undesirable addition of toxic co-surfactants [14]. Consequently, their cytotoxic effect in both cell lines, in the presence and absence of DPS-2, was examined through the thiazolyl blue tetrazolium bromide (MTT) cell proliferation assay. The effective release of a lipophilic compound was evaluated via confocal microscopy and the molecular mechanism of the induced cell death was investigated through fluorescence-activated cell sorting (FACS) analysis, comet assay, and western blotting. The non-small cell lung adenocarcinoma cell line A549, bearing a functional p53 gene (p53^wt^), was also treated under the same conditions and analyzed in parallel to serve as reference with regard to the p53 context.

In the course of the in vitro biological evaluation, O/W microemulsions proved suitable hosts for DPS-2 delivery in WM 164 and Caco-2 cell lines. Moreover, DPS-2 induced S phase delay accompanied by cdc25A degradation as well as a nonapoptotic cell death, implying that this benzothiophene analogue is a deoxyribonucleic acid (DNA) inhibitor.

## 2. Materials and Methods

### 2.1. Materials

Starting materials, and all solvents and reagents for chemical synthesis were purchased from Sigma-Aldrich (Taufkirchen, Germany).

Triacetin (>98%) was purchased from TCI Chemical Industry Co., Ltd. (Tokyo, Japan). Polyoxyethylene sorbitan mono-oleate (Tween 80) suitable for cell cultures and 5-doxyl-stearic acid [5-(1-oxyl-2,2-dimethyl-oxazolidin) stearic acid] (5-DSA), free radical were purchased from Sigma-Aldrich. Nile Red was purchased from Sigma-Aldrich and prepared as a stock solution of 0.2 mg/mL in triacetin.

High-purity water was obtained from a Millipore Milli-Q Plus water purification system.

Phenol red Dulbecco’s modified Eagle medium (DMEM), nonessential amino acids solution (100X), fetal bovine serum (FBS), l-glutamine (200 mM), bovine serum albumin (BSA), trypsin 0.25%, and phosphate-buffered saline (PBS) were purchased from Gibco- Life Technologies (Grand Island, NY, USA). All cell culture plastic ware was supplied by Corning Costar (Lowell, MA, USA). Thiazolyl blue tetrazolium bromide and dimethyl sulfoxide (DMSO) were obtained from Sigma-Aldrich. 

PI/RNase staining solution was purchased from Cell Signaling Technology (Danvers, MA, USA), radio immunoprecipitation assay (RIPA) lysis buffer from Santa Cruz Biotechnology, Inc. (Heidelberg, Germany) and the enhanced chemiluminescence (ECL) Western blotting detection reagent kit from Amersham (Amersham Life Science, Little Chalfont, UK). Antibodies were purchased from Santa Cruz Biotechnology, Inc. (Santa Cruz Biotechnology, Dallas, TX, USA), R&D Systems (Minneapolis, MN, USA), and Cell Signaling Technology (Danvers, MA, USA).

^1^H nuclear magnetic resonance (NMR) spectra were recorded at 300 or 600 MHz on Varian 300 or 600 spectrometer (VARIAN Inc, Palo Alto, California, USA). ^13^C NMR spectra were recorded at 75 MHz on a Varian 300 spectrometer. ^1^H NMR and ^13^C NMR chemical shifts (δ) are reported in parts per million (ppm) relative to residual proton signals in deuterated chloroform (CDCl_3_; δ = 7.26, 77.16 ppm), deuterated methanol (CD_3_OD; δ = 4.87, 49.00 ppm) or deuterated DMSO (DMSO-D_6_; δ = 2.50, 39.43 ppm). Coupling constants (*J*) are reported in Hz and refer to apparent multiplicities. The following abbreviations are used for the multiplicities: singlet (s), doublet (d), triplet (t), quartet (q), multiplet (m), doublet of doublet (dd), doublet of triplet (dt), and broad (br). Melting points (m.p.) were determined on a Buchi 130 capillary melting point apparatus (Buchi, Konstanz, Germany) and are uncorrected. Mass spectra were recorded on a Thermo Fleet instrument (Thermo Fischer Scientific, Inchinnan Bussiness Park, UK) by positive or negative electrospray ionization (ES+ or ES−). High resolution mass spectrometry (HRMS) were recorded on a Thermo Velo instrument (Thermo Fischer Scientific, Inchinnan Bussiness Park, UK). Samples were infused in solution usually at 10–20 μL/min by means of a syringe pump. MeOH was used as solvent. Thin-layer chromatography (TLC) was carried out using Macherey-Nagel silica plates (Macherey-Nagel, Hoeralt, France) with a layer thickness of 0.25 mm SIL G-25 60 UV254. Visualization was effected with ultraviolet (UV) light and phosphomolybdic acid reagent 10% in 95% ethanol. Tetrahydrofurane (THF) was distilled from sodium/benzophenone ketyl.

### 2.2. Synthesis of DPS-2

**Scheme 1:**
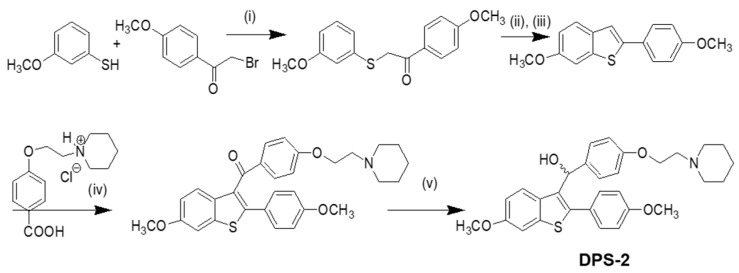
Synthesis of DPS-2. Reagents and conditions: (**i**) KOH, EtOH/H_2_O/EtOAc, 25℃, 18 h, 85%; (**ii**) polyphosphoric acid (PPA), 85 °C, 2 h; (**iii**) acetone, reflux, 1 h, 61% (in two steps); (**iv**) SOCl_2_/AlCl_3_, C_6_H_5_Cl, 75 °C, 2 h, dichloromethane (DCM), 1 h, 25℃, 62%; (**v**) lithium aluminum hydride (LAH), tetrahydrofuran (THF), 0 °C, 45 min, 94%.

Our approach provided the targeted compound DPS-2 in 5 synthetic steps. The key intermediate 6-methoxy-2-2(4-methoxyphenyl)-benzo[*b*]thiophene was prepared by the cyclization-rearrangement produced by polyphosphoric acid (PPA) as described in the literature by Jones and coworkers [21], and depicted in Scheme 1.

The intermediate ether side chain methyl 4-(2-(piperidin-1-yl)ethoxy) benzoic acid hydrochloride was prepared by alkylation of methyl *p*-hydroxybenzoate with 1-(2-chloroethyl) piperidine hydrochloride by means of powdered anhydrous potassium carbonate, followed by base hydrolysis. Friedel–Crafts acylation of 6-methoxy-2-2(4-methoxyphenyl)-benzo[*b*]thiophene with methyl 4-(2-(piperidin-1-yl)-ethoxy) benzoic acid chloride and lithium aluminium hydride (LAH) reduction of the corresponding benzo[*b*]thien-3-yl ketone provided the desired (4-(2-(piperidin-1-yl)ethoxy)phenyl)(6-methoxy-2-(4-methoxyphenyl)benzo[*b*]thiophen-3-yl)methanol (DPS-2). (4-(2-(Piperidin-1-yl)ethoxy)phenyl)(6-methoxy-2-(4-methoxyphenyl)benzo[*b*]thiophen-3-yl)methanone hydrochloride (0.120 g, 0.2088 mmol) was dissolved in tetrahydrofuran (THF;1.2 mL) and the corresponding solution was cooled at 0 °C. Then LAH was added (0.019 g, 0.5 mmoL) and the reaction mixture was stirred at 0 °C for 45 min under argon. After the completion of the reaction (as monitored by TLC), THF (3 mL), THF/H_2_O (4 mL) and H_2_O (4 mL) were added. The product was extracted with methylene chloride (2 × 30 mL) and the organic phase washed with water (10 mL) and saturated solution of sodium chloride (10 mL). It was dried over anhydrous sodium sulfate and concentrated in vacuo. The residue was purified with flash column chromatography using CHCl_3_/MeOH (97:3) as eluent. We obtained 0.1 g, 89% yield, of a light yellow oil which solidified in the freezer. 

Retention factor (Rf), (CHCl_3_/MeOH 9:1) = 0.40 cm

^1^H NMR (300 MHz, CDCl_3_): δ 1.35–1.5 (m, 2H, –C*H*_2_–),1.55–1.65 (m, 4H, –C*H*_2_–), 2.4–2.55 (m, 4H, –NCH_2_–), 2.74 (t, 2H, –CH_2_N–, *J* = 6 Hz), 3.82 (s, 6H, –OC*H*_3_), 4.23 (t, *J* = 6 Hz, 2H, –OC*H*_2_), 6.15 (s, 1H, –HOC*H*), 6.75–6.85 (m, 3H, ArH), 6.87-6.97 (m, 2H, ArH), 7.27 (s, 1H, ArH), 7.30 (d, 2H, ArH, *J* = 8.4 Hz); 7.37–7.47 (m, 2H, ArH), 7.59 (d, 1H, ArH, *J* = 8.4 Hz), ^13^C NMR (75 MHz, CDCl_3_): δ 24.01, 25.64, 54.93, 57.79, 65.64, 69.46, 69.52, 104.67, 113.80, 114.11, 114.33, 125.48, 126.19, 127.44, 130.83, 132.01, 132.52, 134.83, 138.95, 140.72, 157.09, 157.81, 159.71; MS-ESI *m*/*z* 504.41 [M + H]^+^; HRMS *m*/*z* found 504.2199 [M + H]^+^ (calculated for C_30_H_34_NO_4_S 504.2203)

### 2.3. Preparation of the Microemulsions

Oil-in-water microemulsions consisting of triacetin as the oil phase, PBS as the aqueous phase and Tween 80 as surfactant were developed to be used for the delivery of DPS-2. The phase behavior of the microemulsions was determined by a ternary phase diagram (Figure S1) prepared as described elsewhere [14]. The composition of the microemulsions chosen for drug encapsulation was 81.5% *w*/*w* PBS buffer, 10.6% *w*/*w* Tween 80, and 7.9% *w*/*w* triacetin. The particular composition was selected among others, described in ternary phase diagram, containing a small but adequate quantity of Tween 80 and sufficient quantity of oil phase.

### 2.4. Encapsulation of DPS-2

Absolute ethanol was added to solid DPS-2 to form a clear ethanol solution (6.6 mM). Then the appropriate amount of the solution was placed in 1.5 mL tube. After evaporation of the alcohol, an O/W microemulsion consisting of 81.5% *w*/*w* PBS buffer, 10.6% *w*/*w* Tween 80, and 7.9% *w*/*w* triacetin was added in the tube. The system was left overnight in a water bath (25 °C) to allow solubilization of the drug in the oil phase of the microemulsion. The final overall concentration of DPS-2 in the microemulsion was 3.3 mM.

### 2.5. Dynamic Light Scattering

Light scattering measurements were performed using the Zetasizer Nano ZS (Malvern Instruments, Malvern, UK) analyzer equipped with a He-Ne laser (632.8 nm) and noninvasive backscatter optics (NIBS). Detection was carried out in a backscattering mode (scattering angle 173°). The hydrodynamic radii (*R*_h_) of the dispersed oil droplets were obtained from the Stokes–Einstein equation: *R*_h_ = *k_B_T*/6πη*D*, where *k_B_* is the Boltzmann constant, *T* the absolute temperature, η the viscosity of the continuous phase at a given temperature, and *D* is the diffusion constant [22,23].

All components of the microemulsions were filtered separately through either hydrophilic or lipophilic filters (0.45 μm) depending on their polarity. Measurement data were processed using the Malvern Zetasizer Nano software (version 6.32, Malvern Panalytical Ltd, Enigma Business Park, UK) which fits a spherical model of diffusing particles with polydispersity below a value of 0.1. The data are first analyzed by cumulant analysis to obtain an average diffusion coefficient and subsequently by CONTIN analysis in order to obtain information about the entire distribution of the particle size.

Dynamic light scattering (DLS) analysis gives two values, a mean value for the size (intensity mean), and a width parameter known as the polydispersity index (PdI). Size and PdI values of empty and loaded microemulsions were evaluated at 25 °C. All measurements were carried out in triplicate.

### 2.6. Electron Paramagnetic Resonance Spectroscopy

Electron paramagnetic resonance (EPR) measurements were performed at room temperature with a Bruker EMX EPR spectrometer (Bruker, Billerica, MA, USA) operating at the X-band (9.8 GHz). Samples were contained in a quartz EPR flat cell by Wilmad (Buena, NJ). The EPR spectra were recorded with a center field of 0.349 T, scan range 0.01 T, gain of 5.64 × 103, time constant of 5.12 ms, conversion time of 5 ms, modulation amplitude of 0.4 mT, frequency of 9.78 GHz. 5-DSA concentration in the microemulsions was 10–4 M. Oil phase was kept constant 7.9% *w*/*w* throughout the experiment.

Data collection and analysis were performed using the Bruker WinEPR acquisition and processing program (Manufacturer, City, State, Country). All spectral simulations were performed with home-written programs in MATLAB (The MathWorks, Natick, MA, USA) employing the Easy Spin toolbox for EPR spectroscopy [24].

The rotational correlation time (τ_R_) of the spin probes was calculated from the chili function which computes the EPR spectra in the slow-motional regime. The simulation is based on solving the stochastic Liouville equation on a basis of rotational Eigen functions [24].

From the spectral characteristics of the simulated EPR spectra we calculated the order parameter (S) which provides a measure of the spin probe’s arrangement in a supramolecular assembly and varies from 0 to 1, with S = 1 for the completely ordered state and S = 0 for the completely random state. The order parameter, S, is defined as

S = (A_‖_ − A_⊥_)/[A_ZZ_ − 1/2(A_XX_ + A_YY_)]k

where A_XX_, A_YY_, and A_ZZ_ are the single crystal values of the spin probe equal to 6.3, 5.8, and 33.6 Gauss, respectively. A_‖_ and A_⊥_ are the hyperfine splitting constants, where A_‖_ is the half distance of the outermost EPR lines, while A_⊥_ is calculated from the half distance of the inner EPR lines. The ratio k = α_0_/α’_0_ is the polarity correction factor, where α’_0_ = 1/3(A_zz_ + A_xx_ + A_yy_) and α_0_ = 1/3(A_‖_ + 2A⊥). α’_0_ is the isotropic hyperfine splitting constant for the nitroxide molecule in the crystal state and α_0_ is the isotropic hyperfine splitting constant for the spin probe in the membrane [25].

### 2.7. Cell Lines, Culture, and Treatments

The human melanoma cell line WM 164 (BRAF^V600E^, p53^Y220C^; Wistar Institute Melanoma Research Centre, https://wistar.org/) were generously provided by Dr. G. Skrettas (National Hellenic Research Foundation, Athens, Greece) The human colorectal adenocarcinoma cell line Caco-2 (BRAF^V600E^, p53^null^) [15] and non-small cell lung carcinoma cell line A549 (p53^wt^) were purchased from the American Type Culture Collection (ATCC, Manassas, VA, USA).

The human cell lines were grown in DMEM (containing glucose 4.5 g/L, l-glutamine and pyruvate), supplemented with 10% FBS and 1% penicillin/streptomycin (Gibco-Life Technologies), at 37 °C in a humidified incubator with 5% CO_2_. The cells were maintained as a monolayer culture and trypsinized when they reached 70% confluence.

Cells were treated with the bioactive compound either encapsulated in O/W microemulsions or diluted in DMSO at the final concentration of 5.6 μM. The DMSO concentration in the medium did not exceed 0.1% *v*/*v*. Cells were harvested using 0.25% trypsin solution in PBS at 24, 48, 72 and in certain cases 96 h after the treatment onset. Non-treated cells were used in all cases as control.

### 2.8. Cell-Proliferation Assay

Cytotoxicity was initially assessed 24, 48, 72, and 96 h after treatment by MTT assay (M5655; Sigma-Aldrich) according to the manufacturer’s standard protocol. All assays were carried out in triplicate. Statistical analysis was performed by Student’s *t*-test.

### 2.9. Confocal Microscopy

Cells were plated on coverslips at a density of approximately 1.5 × 10^5^ cells per well of a six-well plate and were allowed to attach for 24 h. They were then treated with O/W microemulsions loaded with the lipophilic dye Nile red at a concentration of 0.014 mg/mL for 24, 48, or 72 h, washed with PBS, fixed, with methanol and stained with Alexa Fluor 488 phalloidin (Cell Signaling Technology) against F-actin prior to mounting with Mowiol (Thermo Fischer Scientific, Whaltham, MA, USA). Confocal microscopy was performed on the Leica 626 TCS SPE confocal laser scanning microscope (Leica Microsystems GmbH, Wetzlar, Germany). LAS AF software was used for image 627 acquisition (Leica Lasertechnik, Heidelberg, Germany).

### 2.10. Flow Cytometry

Cancer cell lines were plated at 1.5 × 10^5^ cells per well of a six-well plate and grew overnight followed by 24, 48, and 72 h incubation with O/W microemulsions empty at 0.2% *v*/*v* in culture medium, O/W microemulsions loaded with 5.6 μM DPS-2 or DMSO-diluted DPS-2 at a final concentration of 5.6 μM. After the incubation at the selected time points in cell culture, cells were trypsinized and fixed in ice-cold 100% ethanol overnight at 4 °C. Cells were washed with PBS, stained with propidium iodide for 2 h and subjected to FACS analysis on a FACSCalibur flow cytometer (BD Biosciences, San Jose, CA, USA). Data were analyzed using the CellQuest (BD Biosciences) and ModFit LT software (Verity Software House, Topsham, ME, USA).

### 2.11. Comet assay for Measurement of DNA Damage

Caco-2 cells (2 × 10^5^ cells/well) were placed in six-well plates for 24 h and then were incubated with O/W microemulsions empty at 0.2% *v*/*v* in culture medium and DPS-2 encapsulated as well as diluted in DMSO, at a concentration of 5.6 μM for 48 h. Cells were examined for DNA damage as previously described [26,27]. Images were obtained at ×400 magnification using a fluorescence microscope. From each slide >100 cells were selected and photographed randomly. The percentage of DNA in the tail, relative to the head, was calculated using ImageJ OpenComet software [28]. Statistical analysis was performed with nonparametric Mann–Whitney rank sum test.

### 2.12. Western Blotting

Total protein extracts were prepared using RIPA lysis buffer. Cell lysates (25–40 μg protein) were resolved by sodium dodecyl sulfate polyacrylamide gel electrophoresis (SDS–PAGE) gel electrophoresis, electrotransferred onto nitrocellulose (0.2 μm) and blocked as previously described [17]. Overnight incubation of the nitrocellulose with primary antibody at 4 °C was followed by incubation with secondary antibody conjugated to horseradish peroxidase (HAF008; R&D Systems) for 1.5 h at room temperature. Detection was achieved by ECL Western blotting detection reagent kit. Primary antibodies against the following proteins were used at the indicated dilutions: PARP (sc-7150; Santa Cruz Biotechnology), 1:500; cdc25A (sc-97; Santa Cruz Biotechnology), 1:200; β-actin (MAB8929; R&D Systems), 1:1000.

## 3. Results

### 3.1. Structural Study of the Microemulsions

#### 3.1.1. Dynamic Light Scattering Measurements

**Figure 2:**
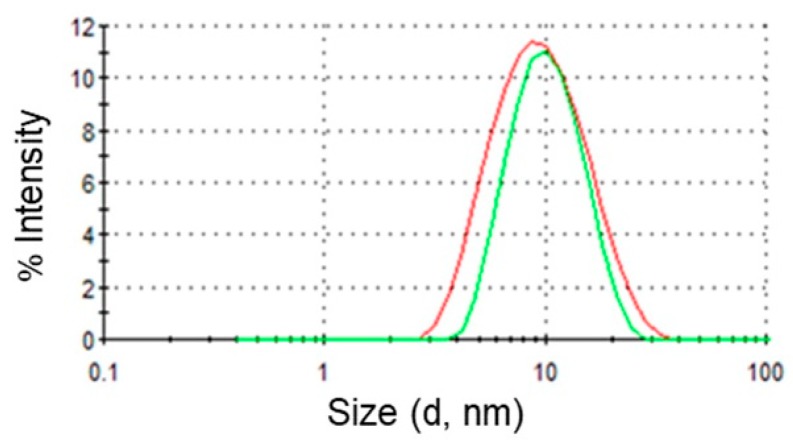
Intensity distribution of droplet sizes. Red line: empty O/W microemulsions; green line: O/W microemulsions loaded with DPS-2.

Dynamic light scattering measurements of O/W microemulsions were carried out to evaluate the size and the polydispersity of the dispersed oil droplets in the presence and in the absence of DPS-2. Figure 2 represents distribution functions of the hydrodynamic radii of the two microemulsions. As can be clearly observed, addition of the bioactive molecule in the oil cores of the microemulsions did not affect their size. In particular, the mean droplet diameter of dispersed oil droplets was 10 ± 0.1 nm for the empty and 10 ± 0.4 nm for the full systems. Nevertheless, a small increase of the polydispersity index from 0.17 ± 0.02 to 0.27 ± 0.03 was observed upon DPS-2 addition.

#### 3.1.2. Electron Paramagnetic Resonance Spectroscopy

**Figure 3:**
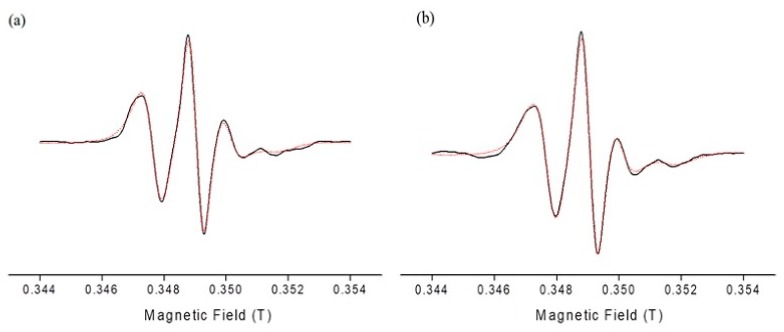
Electron paramagnetic resonance (EPR) spectra of 5-DSA in (**a**) empty and (**b**) loaded with DPS-2 O/W microemulsions. Black line: experimental spectrum; red dotted line: simulated spectrum.

Electron paramagnetic resonance spectroscopy using the spin-probing technique was undertaken to study interfacial properties of the surfactants’ monolayer in both empty and DPS-2 loaded O/W microemulsions. The spin-labelled fatty acid, 5-DSA, which was used as spin probe, is a long amphiphilic molecule consisting of stearic acid and an *N*-oxyl radical (doxyl group) directly attached to the C-5 position of the hydrocarbon chain. When 5-DSA is solubilized in microemulsions, it is localized at the interfaces interacting with the surfactant molecules. Information reflecting the rigidity/flexibility of their environment from the depth of the membrane where the doxyl ring is located can be obtained. More specifically, in the case of 5-DSA, the nitroxide is located closer to the polar head of the amphiphilic fatty acid and consequently closer to the surfactant polar heads. The EPR spectra from the spin-labelled fatty acid in empty and loaded O/W microemulsions were obtained and analyzed using an anisotropic rotational model based on the slow-motion theory developed by Schneider and Freed [29]. Figure 3 shows experimental and simulated EPR spectrum of 5-DSA in empty and loaded microemulsions. In general, EPR spectra of unequal heights and widths are indicative of a restrictive motion of the spin probe in the membrane where it is located.

Then, EPR spectra were analyzed to obtain information regarding molecular motion and order in the surfactants layer. Rotational correlation times τ_R_ and order parameters S calculated from the simulated EPR spectra are shown in Table 1.

As it can be observed, by adding DPS-2 in the oil cores of the microemulsions rotational correlation time τ_R_ of 5-DSA was not affected since the observed decrease from 6.01 ± 0.20 to 5.95 ± 0.19 cannot be evaluated as indicative of structural changes. Table 1 also shows the order parameters S for drug free and drug containing microemulsions at constant oil and surfactant concentrations. At oil phase weight fraction equal 7.9%, both in the presence and in the absence of DPS-2, the system is characterized by high order parameters S of 0.45 and 0.48, respectively, indicating a very restricted movement of the spin probe in the interface. The observed small increase of order parameter upon drug encapsulation clearly excludes drug participation in the surfactants monolayer.

### 3.2. Biological Assessment

In order to evaluate the O/W microemulsion system in terms of drug delivery and cytotoxicity, we applied the following in vitro approaches in the human MW 164 skin melanoma and Caco-2 epithelial colorectal adenocarcinoma cell lines.

#### 3.2.1. Confocal Microscopy

**Figure 4:**
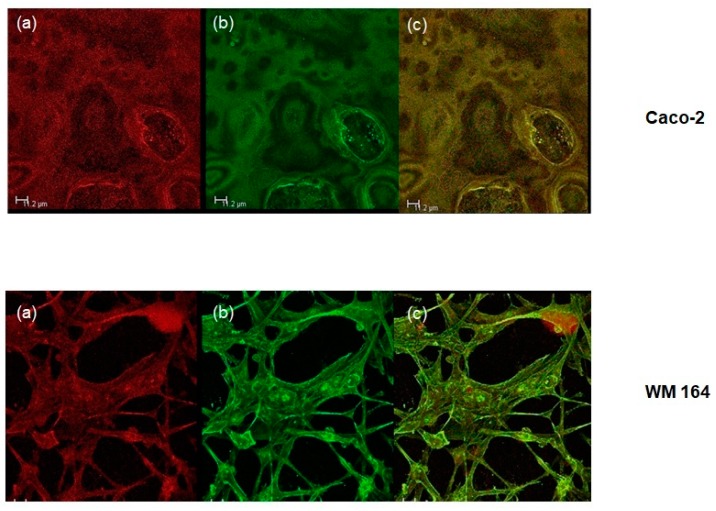
Colocalization of the oil phase with the cytoskeleton in both cell lines tested at 72 h. (**a**) Red: Nile Red staininglabels the oil phase; (**b**) Green: fluorescent secondary antibody against an anti-β-actin antibody labels the cytoskeleton; (**c**) Yellow: merged. Scale bar: 11.2 μm.

The intracellular release of a lipophilic compound through the O/W microemulsion structure was assessed through confocal microscopy. Cells were prepared as described in the Materials and Methods Section. The oil phase was labelled with Nile Red while the cytoskeleton of both cells lines was labeled with a green fluorescent secondary antibody against the anti-β-actin antibody. The intracellular delivery of the oil phase was followed for 3, 6, 24, 48, and 72 h. The oil phase colocalized with the cytoskeleton already at 6 h of treatment and remained as such as long as 72 h (Figure 4). 

#### 3.2.2. Cell Proliferation Assay

**Figure 5:**
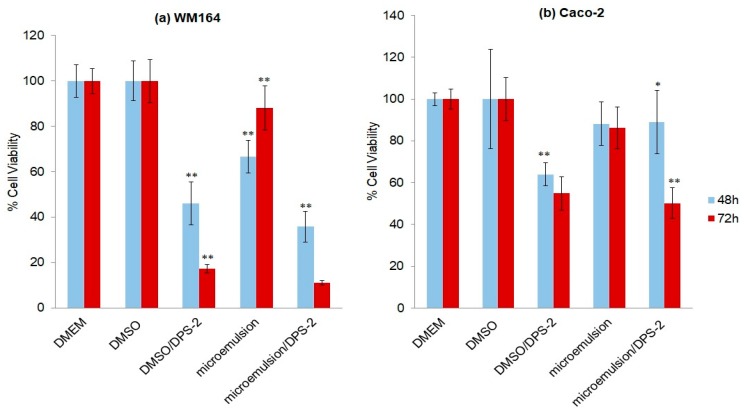
Thiazolyl blue tetrazolium bromide (MTT) cell proliferation assay in (**a**) WM 164 and (**b**) Caco-2 cell lines after 48 and 72 h of treatment: Dulbecco’s modified Eagle medium (DMEM); dimethyl sulfoxide (DMSO) 0.1% *v*/*v*; DPS-2 solubilized in DMSO at 5.6 μM, as positive control samples; O/W microemulsion empty 0.2% *v*/*v*; O/W microemulsion loaded with DPS-2 at 5.6 μM. The mean (± SD) of three independent experiments, each performed in five replicates, is presented. Statistically significant results are indicated by asterisks (*) when *p* < 0.05 and (**) when *p* < 0.01 (Student’s *t*-test).

Cell proliferation assays were performed using the MTT assay, a method for sensitive quantification of viable cells, in the above-mentioned cell lines, both bearing the BRAF^V600E^ mutation and a nonfunctional p53 [15,16]. Cells were treated with the lipophilic compound DPS-2 either diluted in DMSO or encapsulated in O/W microemulsions. The effect of microemulsions in the absence and presence of the lipophilic compound was determined in comparison to its effect when dissolved in DMSO. The concentration of the compound dissolved in DMSO is equal to the concentration encapsulated in the microemulsions. Microemulsions were administered into the cell culture following dilution at a final ratio of 0.2% or 0.5% *v*/*v* in the culture medium, because within this range the nanocarrier does not exhibit any cytotoxicity [14]. The percentage of cell viability 48 and 72 h after treatment is shown in Figure 5 (DMEM and DMEM plus DMSO at 0.1% *v*/*v* were used as control samples). As observed, O/W microemulsions empty at a final ratio of 0.2% *v*/*v* in the medium, did not inhibit cell proliferation in either cell line. On the contrary, O/W microemulsions loaded with the lipophilic compound significantly inhibited cell proliferation, the cytotoxic effect being prominent at 72 h in both cell lines. The effect was further enhanced in Caco-2 cells when treatment was kept as long as 96 h (Supplementary Figure S2). For reasons of comparison, cell proliferation assays were in parallel applied in the non-small cell lung carcinoma cell line A549 (p53^wt^) treated under the same conditions and at the same time points; A549 cells were resistant to the cytotoxic effects of DPS-2 and, despite their shorter doubling time (22 h), a marginal (≈10%), nonstatistically significant inhibition of cell proliferation was observed at 72 h (Supplementary Figure S3).

Overall, these findings indicate the suitability of the proposed O/W microemulsions as carriers of the lipophilic compound DPS-2 into human cell lines. Importantly, encapsulation of DPS-2 in the oil cores of the microemulsions did not impose increased cytotoxicity as compared to the conventional administration using DMSO.

#### 3.2.3. Cell Cycle Analysis through Propidium Iodide Staining

**Figure 6:**
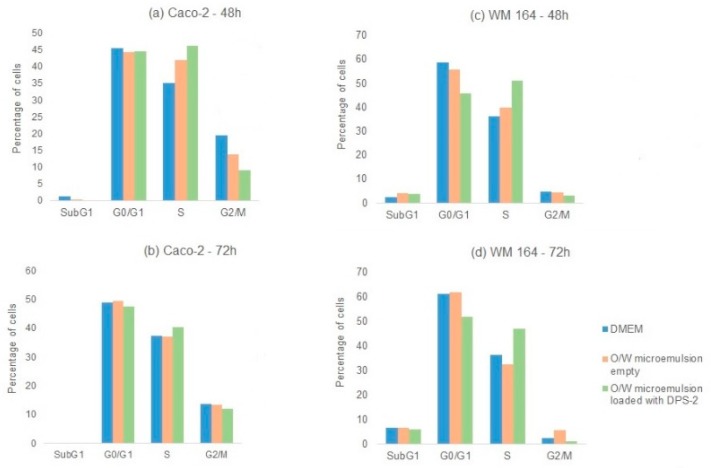
Cell cycle analysis. Non-treated (DMEM) and treated (O/W microemulsion empty 0.2% *v*/*v*; O/W microemulsion loaded with DPS-2 at 5.6 μM) (**a**) WM 164 and (**b**) Caco-2 cells harvested at 48 and 72 h (triplicates were pooled) were stained with propidium iodide and subjected to cytofluorometric analysis of deoxyribonucleic acid (DNA) content. A representative experiment out of two similar ones is depicted.

Cytofluorometric analysis of DNA content was performed in parallel in WM 164 and Caco-2 cells. The benzothiophene-based DPS-2 solubilized in DMSO, at the same concentration (5.6 μM), was also administered in both cell lines and samples were analyzed. Cells were again harvested and analyzed at 24, 48, and 72 h after treatment. No significant increase in the sub-G1 phase was observed in any case, indicating an alternative nonapoptotic cell death mechanism. As shown in Figure 6, in both cases DPS-2 treatment induced S phase delay. Cell cycle analysis of A549 cells (p53^wt^) upon treatment under the same conditions and at the same time points was again performed in parallel; in this case, DPS-2 treatment induced G1 arrest at 72 h, regardless the means of DPS-2 administration (Supplementary Figure S4).

Overall, these results indicate that in a nonfunctional p53 cell context, DPS-2 induced inhibition of cell proliferation and cell death through S phase arrest, which implies attack at the DNA replication fork level.

#### 3.2.4. Comet Assay

**Figure 7:**
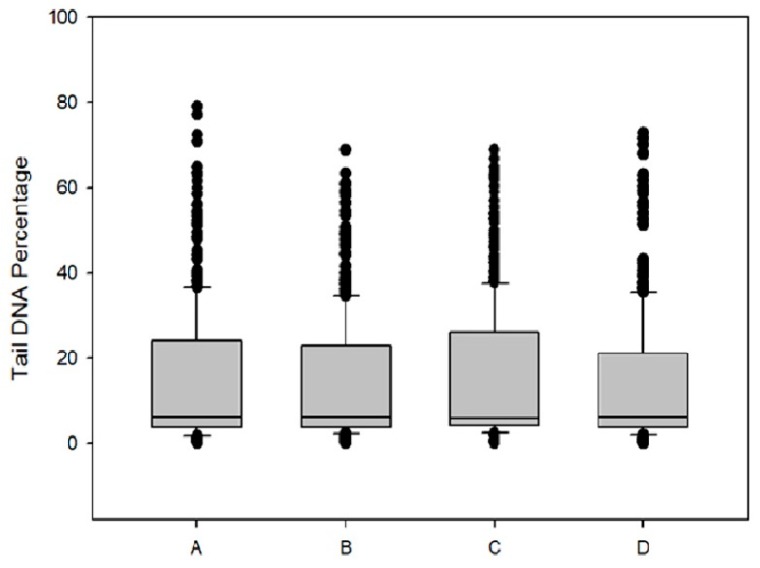
Box plots of tail DNA percentage of Caco-2 cells after 48h of DPS-2 treatment: (**A**) DMEM; (**B**) DPS-2 solubilized in DMSO at 5.6 μM; (**C**) O/W microemulsion empty 0.2% *v*/*v*; (**D**) O/W microemulsion loaded with DPS-2 at 5.6 μM.

Genotoxicity of DPS-2 was investigated in Caco-2 cells after 48 h treatment. As depicted in Figure 7 by the tail DNA percentage diagram, no DNA damage was detected indicating that DPS-2 is not a direct genotoxic agent.

#### 3.2.5. Molecular Analysis by Western Blotting

**Figure 8:**
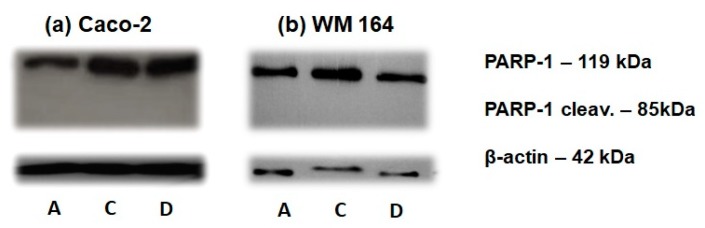
Cleavage of poly(adenosine diphosphate-ribose) polymerase 1 (PARP-1) for the assessment of cell death. Immunoblot of (**a**) Caco-2 and (**b**) WM 164 cell extracts (40 μg) with anti-PARP-1 antibody detecting the 85 kDa PARP fragment (cleav. PARP-1) and the 119 kDa intact PARP-1 form. Cells were either not treated (DMEM (A)), or treated with O/W microemulsion empty 0.2% *v*/*v* (C) or O/W microemulsion loaded with DPS-2 at 5.6 μM (D) (and maintained in culture for 72 h. The relative abundance of total protein applied was measured by using as control the amount of actin as assessed by anti-β-actin on the same blot.

In an effort to start investigating the molecular mechanism of the induced cell death, biochemical analysis of DNA replication block and cell death markers was performed by Western blotting. The specific cleavage of poly(adenosine diphosphate-ribose) polymerase 1 (PARP-1), a nuclear protein implicated in DNA repair, to a 89 kDa fragment as a result of the caspase protease activity associated with activation of apoptosis, is considered as a sensitive apoptotic marker [17]. As shown in Figure 8, PARP-1 cleavage was not detected in either cell line following DPS-2 treatment, in accordance with the lack of sub-G1 phase in cell cycle analysis.

**Figure 9:**
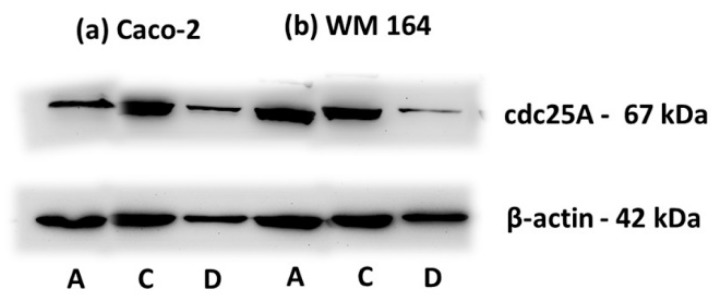
Immunodetection of cdc25a in protein extracts of (**a**) Caco-2 and (**b**) WM 164 cells 72 h after treatment. Cells were either not treated (DMEM (A)), or treated with O/W microemulsion empty 0.2% *v*/*v* (C) or O/W microemulsion loaded with DPS-2 at 5.6 μM (D) and maintained in culture for 72 h. All extracts (25 μg) were immunoblotted with anti-cdc25a antibody detecting the 67 kDa protein. The relative abundance of total protein applied was measured by using as control the amount of actin as assessed by anti-β-actin on the same blot (42 kDa).

Furthermore, the biochemical changes of cdc25A, a member of the cdc25 family of dual-specificity protein phosphatases acting as positive regulators of the cell division cycle, were analyzed. cdc25A protein levels are tightly regulated and remain low throughout interphase, rise during mitosis while the protein itself is rapidly destroyed when cells are exposed to agents that hinder DNA replication. It is required for progression from G1 to S and thus considered as a specific S phase arrest marker [30,31]. As clearly shown in Figure 9, a degradation of cdc25A, in accordance with the S phase arrest indicated in the course of cell cycle analysis, was observed in WM 164 and Caco-2 cells. The biochemical analysis confirmed the lack of apoptosis and induction of cell death through S phase arrest, implying that DPS-2 directly inhibits DNA replication and thus cell proliferation, at the replication fork level.

## 4. Discussion

Pharmaceutically applicable microemulsions based on Tween 80, triacetin and buffer solution were developed as matrices for the encapsulation of DPS-2, a novel benzothiophene analogue. Triacetin, also known as glyceryl triacetate, was selected as the oil phase of the microemulsions since it has been generally recognized as safe (GRAS) human food ingredient, nontoxic to animals in acute oral or dermal exposures and in short-term inhalation or parenteral studies [32]. On the other hand, Tween 80 used as surfactant is a well-accepted nonionic amphiphile, widely used in many pharmaceutical formulations.

Microemulsions, being nanostructured vehicles with different domains of variable polarity are considered as promising formulations, particularly useful in the area of drug delivery. To thoroughly understand the drug delivery potential of the microemulsions under investigation, it is necessary to know their structural characteristics and also the influence of the drug on the microstructure. Therefore, the microemulsions developed in this study were subjected to two different analytical techniques, DLS and EPR spectroscopy. In general, structural information obtained from EPR studies could be of importance in the perspective of using colloidal systems as drug carriers since rigidity of the surfactants monolayer has been related to the permeability and bioavailability of the enclosed bioactive compounds [33].

The observed similarity in τ_R_ and S values indicated that DPS-2 located within the oily cores of the microemulsions and did not interact with the surfactants layer. Interestingly, the increased rigidity of the interfacial film as reflected by the order parameter values and the EPR spectra (Table 1 and Figure 3) did not prevent the release of the encapsulated bioactive molecule. Similar behavior has been reported for PLX4720, a lipophilic antitumor drug, upon encapsulation in similar microemulsions [14].

Finally, DLS was used to determine droplet sizes in empty and drug loaded microemulsions. This technique measures the diffusion of particles moving under Brownian motion, and converts this to size and a size distribution using the Stokes–Einstein relationship. The existence of one population of oil droplets of about 10 nm in diameter was evidenced. Upon addition of DPS-2 in the oil phase the same monomodal particle size distribution was observed verifying the drug’s location in the oil droplets of the O/W microemulsions.

During the last five years, researchers have reported promising results in developing pharmaceutically accepted O/W microemulsions as delivery systems of lipophilic drugs for oral or topical administration. In particular, selective delivery of chemotherapeutics to cancer cells, a major issue in cancer research worldwide, requires the development of effective biocompatible nanocarriers such as microemulsion formulations [1,33]. Cancer involves uncontrolled cell division, replicative immortality, and resistance to cell death. Significant progress in the cancer treatment field has been made, nevertheless, the indiscriminate destruction of cells and the toxic side effects of the chemotherapeutic agents remain a major issue, especially in the treatment of metastatic cancers [34]. During the last two decades, the discovery of the cell signaling networks involved in the control of cell proliferation and differentiation, paved the way to targeted therapy through the design of drugs specifically affecting those networks [20]. Targeted treatments are aimed to block specific transduction pathways or cancer proteins involved in tumor growth and progression, attempting in parallel to deliver the chemotherapeutic agents specifically to cancer cells, in order to minimize the undesirable side effects related to the death of normal cells [20,35]. Therefore, the elucidation of the molecular mechanism underlying the cytotoxic effect of a compound against cancer cells is of paramount importance for the development of targeted, effective therapeutics.

In this context, the investigation of the molecular mechanism underlying the cytotoxic effect of the benzothiophene analogue DPS-2, figuring as a potential lead compound for the development of a novel chemotherapeutic drug, is imperative. So far, due to the wide range of biological activities of the benzothiophene core structure, numerous benzothiophene-based compounds have been developed as lead molecules and further used in the form of clinical drugs, with high therapeutic efficiency, to treat various types of diseases [9,10].

Hence, following the structural characterization, the developed microemulsions, empty as well as loaded with DPS-2, were submitted to in vitro biological evaluation in MW 164 skin melanoma and Caco-2 human epithelial colorectal adenocarcinoma cell lines. The choice of these cancer cell lines is justified by the use of microemulsions for transdermal and oral drug delivery [1,36,37,38]. The oil cores of the O/W microemulsions developed were proven effective carriers of lipophilic compounds in the cell lines examined (Figure 4). Furthermore, DPS-2 encapsulated at a concentration of 5.6 μM, significantly inhibited cell proliferation of the MW 164 skin melanoma and Caco-2 human epithelial colorectal adenocarcinoma cells (Figure 5). Both cell lines were also treated in parallel with DPS-2 solubilized in DMSO at 5.6 μM (Figure 5). Although the O/W microemulsions empty transiently inhibited cell proliferation, the process totally recovered after 48 h as shown in Figure 5, indicating DNA replication checkpoint recovery and replication fork restart [39]. In MW 164 skin melanoma cells the cytotoxic effects of DPS-2, regardless the means of administration, became prominent earlier (48 h, Figure 5a) very likely, reflecting the difference in each cell line’s doubling time (48 h in WM 164 vs. 62 h in Caco-2). In both cell lines, the cytotoxic effect was prominent at 72 h and further enhanced in Caco-2 cells when treatment was kept as long as 96 h (Supplementary Figure S2). Our results are in accordance with the high cytotoxic potential of this benzothiophene analogue in human cancer cell lines observed when administered via DMSO [14].

In cell cycle analysis, DPS-2 induced S phase delay 48 h after treatment (Figure 6) while cdc25A degradation was detected through Western blotting at 72 h in both cancer cell lines (Figure 9). It seems that DPS-2 is a potent cytotoxic agent, capable of stalling the DNA replication fork, directly activating the replication S phase checkpoint which is independent of p53 [39]. However, as confirmed by the comet assay results (Figure 7), DPS-2 is not a direct genotoxic agent. The DNA replication or S phase checkpoint monitors the integrity of DNA synthesis and perturbations in DNA synthesis lead to replication fork stalling and activation of the checkpoint pathway [40,41,42]. If checkpoint recovery mechanisms fail, stalled forks can persist, increasing the likelihood of DNA damage [42]. Based on our results, we assume that as stalled forks persist single-strand breaks (SSBs) and double-strand breaks (DSBs) are finally formed further enhancing the agent’s cytotoxicity which is in agreement with the cell proliferation assay results mentioned above. The destruction of cdc25A in response to genotoxic stress, is proteasome-dependent and independent of the p53–p21 axis [30]. The degradation of cdc25A observed at 72 h of DPS-2 treatment (Figure 9) follows S phase disturbance prominent at 48 h, which along with cytotoxicity results overall supports our hypothesis about secondary induction of DNA damage as a result of DNA replication fork stalling and collapse. It is worth mentioning that exactly the same results were observed when DPS-2, at the same concentration (5.6 μM), was administered via DMSO and samples were analyzed in parallel.

As the cell lines used in this study are p53^null^, in order to investigate the role of functional p53 in the cytotoxic effect of DPS-2, we treated the A549 (p53^wt^) non-small cell lung adenocarcinoma cell line with DPS-2 under the same conditions and performed cell proliferation assay and cell cycle analysis. DPS-2 affected the G1 phase at 72 h of treatment (Supplementary Figure S4), nevertheless, only a marginal, not statistically significant, increase of cytotoxicity was observed at the same time point (Supplementary Figure S3) as functional p53 seems to support DNA replication processivity, preserving DNA integrity before damage occurs [43].

The cell death induced by DPS-2 in WM 164 and Caco-2 cell lines was not apoptotic as indicated by the lack of sub-G1 phase increase in cell cycle analysis (Figure 6) and confirmed by immunoblotting of PARP-1 cleavage (Figure 8). Our findings, overall, indicate replication fork collapse due to DPS-2 treatment, still, further investigation is needed to understand how the compound affects the signaling pathways involved in cellular survival and death, in particular in view of DPS-2’s development as a novel chemotherapeutic drug. Chemotherapeutic agents may activate various modes of cell death and, in general, the cell death pathways finally activated following the interplay of pro-survival and pro-death signaling depend on the cellular background [17,44]. In cancer cell systems, the evasion of cell death being one of the major hallmarks, this interplay becomes even more complicated [20]. Therefore, further investigation of the molecular mechanism underlying the cytotoxic effect of DPS-2 in vitro as well as in vivo is currently in progress as this is of paramount importance for the development of such a compound into a targeted, effective chemotherapeutic agent.

Importantly, the compound encapsulated was as effective as when dissolved in DMSO, a common organic solvent used for administration of chemical agents in cell lines. Although common, DMSO shows certain limitations as it has been reported to exhibit a toxic effect on cultured cells after a particular concentration [45]. In this respect, the oil cores of O/W microemulsions could replace DMSO as a more effective, biocompatible drug carrier when performing biological assessment experiments [1,6,45]. Microemulsions, in general, provide several potential advantages as drug delivery systems since they offer all the possible requirements of a liquid system (thermodynamic stability, easy formation, low viscosity) and also all the advantages deriving from being heterogeneous at the microscopic scale (high surface area, high solubilization capacity, and very small droplet size).

## 5. Conclusions

1. The O/W microemulsions developed here are efficient matrices for the encapsulation of lipophilic bioactive molecules. When loaded, they successfully deliver the compound as far as the nucleus without affecting the functional integrity of the cellular membrane.
2. The proposed O/W microemulsions are suitable as carriers of the lipophilic benzothiophene analogue DPS-2 into human cancer cell lines. Importantly, encapsulation of DPS-2 in the oil cores of the microemulsions did not impose increased cytotoxicity as compared to the conventional administration using DMSO.
3. Due to its potent cytotoxic activity in various human cancer cell lines, DPS-2 is currently being developed as a new lead compound in drug design. This perspective is further confirmed by our results indicating that DPS-2 exerts its cytotoxic activity through stalling and finally collapse of the replication fork.

## Figures and Tables

**Table 1:** Rotational correlation time (τ_R_) and order parameter (S) of 5-DSA in empty and loaded microemulsions.

System	τ_R_ (ns)	S
O/W microemulsion, empty	6.01 ± 0.20	0.45 ± 0.02
O/W microemulsion, loaded	5.95 ± 0.19	0.48 ± 0.02

DPS-2 concentration was 3.3 mM. Experiments were performed in triplicate and the results are expressed as mean values ± standard deviation (SD).

## Supplementary Materials

The following are available online at http://www.mdpi.com/2313-7673/3/2/13/s1. Figures S1–S4. Figure S1: The ternary phase diagram of O/W microemulsion consists of PBS buffer as continuous aqueous phase, triacetin as oil phase and Tween 80 as surfactant, Figure S2: MTT cell proliferation assay in WM 164 and Caco-2 cell lines after 96 h of treatment, Figure S3: MTT cell proliferation assay A549 cell line after 72 h of treatment, Figure S4: Cell cycle analysis.
