# Addendum: Oil-In-Water Microemulsions as Hosts for Benzothiophene-Based Cytotoxic Compounds: An Effective Combination. *Biomimetics* 2018, *3*, 13

**DOI:** 10.3390/biomimetics3040033

**Published:** 2018-10-25

**Authors:** Ioanna Theochari, Vassiliki Papadimitriou, Demetris Papahatjis, Nikos Assimomytis, Efthimia Pappou, Harris Pratsinis, Aristotelis Xenakis, Vasiliki Pletsa

**Affiliations:** 1 Institute of Biology, Medicinal Chemistry & Biotechnology, National Hellenic Research Foundation, 48 Vassileos Constantinou Avenue, 11635 Athens, Greece; jtheohari@eie.gr (I.T.); vpapa@eie.gr (V.Pa.); dpapah@eie.gr (D.P.); nassim@eie.gr (N.A.); epappou@eie.gr (E.P.); arisx@eie.gr (A.X.); 2 Department of Biochemistry and Biotechnology, School of Health Sciences, University of Thessaly, Viopolis, 41500 Larissa, Greece; 3 Laboratory of Cell Proliferation and Ageing, Institute of Biosciences and Applications, National Centre of Scientific Research “Demokritos”, 11635 Athens, Greece; hprats@bio.demokritos.gr

It was brought to our attention that due to recent changes in the regulation that governs the Ph.D. program at the University of Thessaly, it is mandatory to state the academic institution the Ph.D. student is affiliated with. This was omitted in [1], and we would like to add the following information to the affiliation line for the first author, Ioanna Theochari: “Department of Biochemistry and Biotechnology, School of Health Sciences, University of Thessaly, Viopolis, 41500 Larissa, Greece”. 

Line numbers within the affiliation section have been rearranged and changed as follows:

Ioanna Theochari ^1^, Vassiliki Papadimitriou ^1^, Demetris Papahatjis ^1^, Nikos Assimomytis ^1^, Efthimia Pappou ^1^, Harris Pratsinis ^2^, Aristotelis Xenakis ^1^ and Vasiliki Pletsa ^1,^*

^1^ Institute of Biology, Medicinal Chemistry & Biotechnology, National Hellenic Research Foundation, 48 Vassileos Constantinou Avenue, 11635 Athens, Greece; jtheohari@eie.gr (I.T.); vpapa@eie.gr (V.Pa.); dpapah@eie.gr (D.P.); nassim@eie.gr (N.A.); epappou@eie.gr (E.P.); arisx@eie.gr (A.X.)

^2^ Laboratory of Cell Proliferation and Ageing, Institute of Biosciences and Applications, National Centre of Scientific Research “Demokritos”, 11635 Athens, Greece; hprats@bio.demokritos.gr

* Correspodence: vpletsa@eie.gr; Tel.: +302-107-273-7541

has been corrected to

Ioanna Theochari ^1,2^, Vassiliki Papadimitriou ^1^, Demetris Papahatjis ^1^, Nikos Assimomytis ^1^, Efthimia Pappou ^1^, Harris Pratsinis ^3^, Aristotelis Xenakis ^1^ and Vasiliki Pletsa ^1,^*

^1^ Institute of Biology, Medicinal Chemistry & Biotechnology, National Hellenic Research Foundation, 48 Vassileos Constantinou Avenue, 11635 Athens, Greece; jtheohari@eie.gr (I.T.); vpapa@eie.gr(V.Pa.); dpapah@eie.gr (D.P.); nassim@eie.gr (N.A.); epappou@eie.gr (E.P.); arisx@eie.gr (A.X.)

^2^ Department of Biochemistry and Biotechnology, School of Health Sciences, University of Thessaly, Viopolis, 41500 Larissa, Greece

^3^ Laboratory of Cell Proliferation and Ageing, Institute of Biosciences and Applications, National Centre of Scientific Research “Demokritos”, 11635 Athens, Greece; hprats@bio.demokritos.gr

* Correspodence: vpletsa@eie.gr; Tel.: +302-107-273-7541

We apologize for any inconvenience this may have caused. The change does not affect the scientific results. The manuscript will be updated and the original will remain online on the article website, with a reference to this addendum.
