# Supplementary material for: Oil-In-Water Microemulsions as Hosts for Benzothiophene-Based Cytotoxic Compounds: An Effective Combination
Source: Biomimetics (Basel). 2018 Jun 16;3(2):13. doi: 10.3390/biomimetics3020013 (PMC6352693; doi:10.3390/biomimetics3020013)
Supplement: Supplementary file 1 [file biomimetics-03-00013-s001.pdf]

Biomimetics **2018**, *3*, 13

Supplementary Materials: Oil-In-Water Microemulsions as Hosts for Benzothiophene-Based Cytotoxic Compounds: An Effective Combination

Ioanna Theochari [1], Vassiliki Papadimitriou [1], Demetris Papahatjis [1], Nikos Assimomytis [1], Efthimia Pappou [1], Harris Pratsinis [2], Aristotelis Xenakis [1] and Vasiliki Pletsa [1,*]

[1] Institute of Biology, Medicinal Chemistry & Biotechnology, National Hellenic Research Foundation, 48 Vassileos Constantinou Avenue, 11635 Athens, Greece; jtheohari@eie.gr (I.T.); vpapa@eie.gr (V.Pa.); dpapah@eie.gr (D.P.); nassim@eie.gr (N.A.); epappou@eie.gr (E.P.); arisx@eie.gr (A.X.)

[2] Laboratory of Cell Proliferation and Ageing, Institute of Biosciences and Applications, National Centre of Scientific Research "Demokritos", 11635 Athens, Greece; hprats@bio.demokritos.gr

* Correspodence: vpletsa@eie.gr; Tel.: +302-107-273-7541

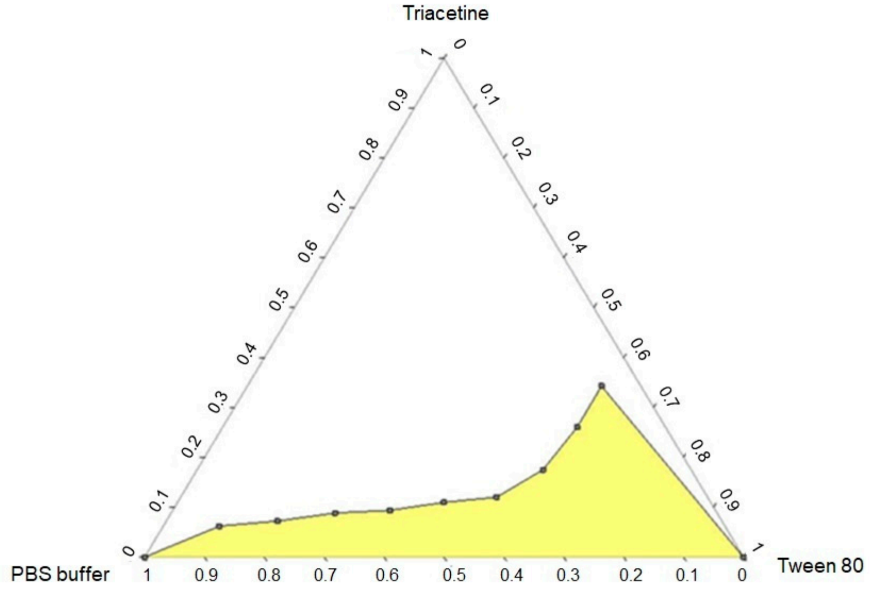

Figure S1. The ternary phase diagram of O/W microemulsion consists of PBS buffer as continuous aqueous phase, triacetin as oil phase and Tween 80 as surfactant.

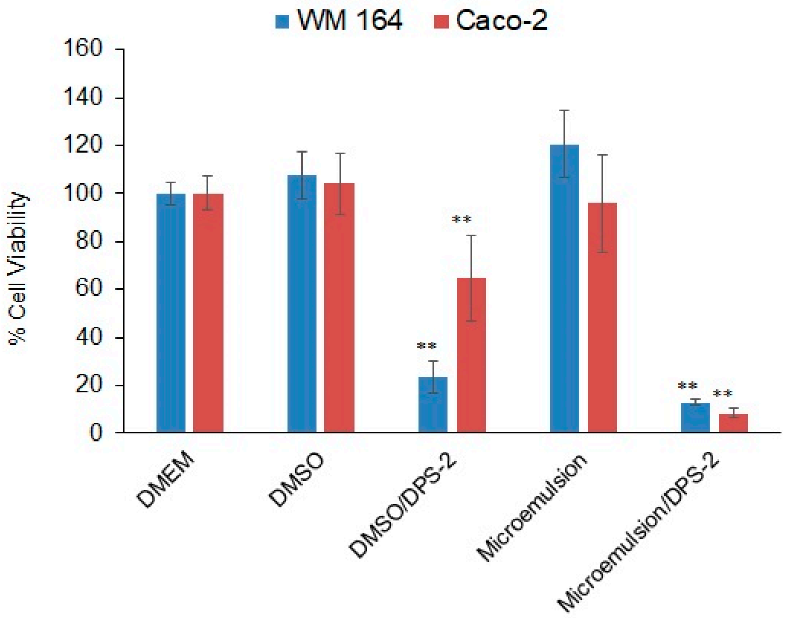

Figure S2. MTT cell proliferation assay in WM 164 and Caco-2 cell lines after 96 h of treatment: DMEM; DMSO 0.1% v/v; DPS-2 solubilized in DMSO at 5.6 µM, as positive control samples; O/W microemulsion empty 0.2% v/v; O/W microemulsion loaded with DPS-2 at 5.6 µM. The mean (±SD) of three independent experiments, each performed in five replicates, is presented. Statistically significant results are indicated by asterisks (*) when $p < 0.05$ and (**) when $p < 0.01$ (Student's t-test).

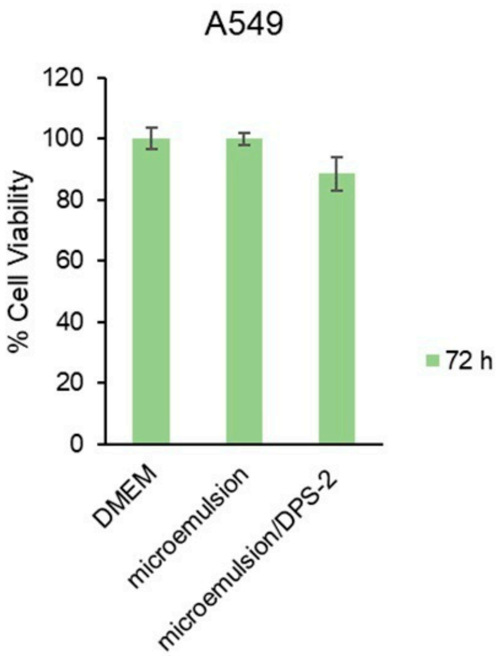

Figure S3. MTT cell proliferation assay A549 cell line after 72 h of treatment: DMEM as positive control sample; O/W microemulsion empty 0.2% v/v; O/W microemulsion loaded with DPS-2 at 5.6µM. The mean (±SD) of three independent experiments, each performed in five replicates, is presented. The results were not statistically significant (Student's t-test).

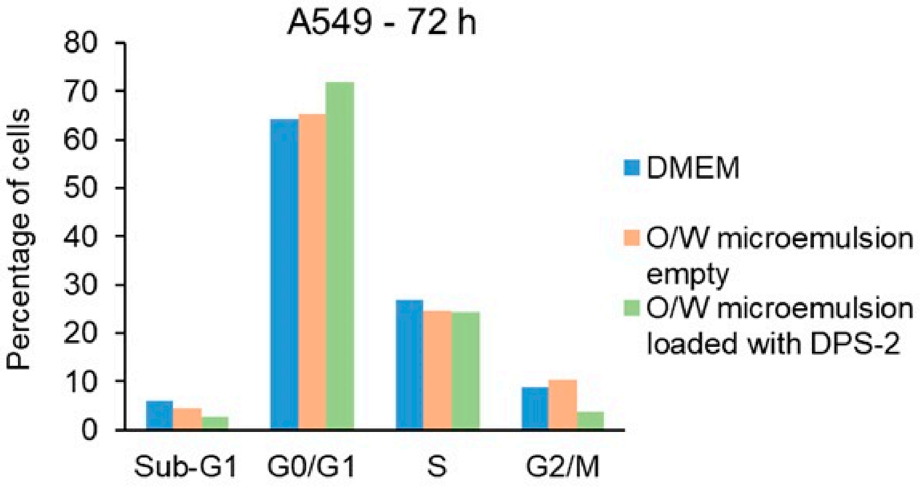

Figure S4. Cell cycle analysis. Nontreated (DMEM) and treated (O/W microemulsion empty 0.2% v/v; O/W microemulsion loaded with DPS-2 at 5.6μM) A549 cells harvested at 72 h were stained with propidium iodide and subjected to cytofluorometric analysis of DNA content. A representative experiment out of two similar ones is depicted.
